# An Alpha-1A Adrenergic Receptor Agonist Prevents Acute Doxorubicin Cardiomyopathy in Male Mice

**DOI:** 10.1371/journal.pone.0168409

**Published:** 2017-01-12

**Authors:** Megan D. Montgomery, Trevor Chan, Philip M. Swigart, Bat-erdene Myagmar, Rajesh Dash, Paul C. Simpson

**Affiliations:** 1 Department of Medicine, Cardiology Division, VA Medical Center, San Francisco, CA, United States of America; 2 Department of Medicine and Cardiovascular Research Institute, University of California, San Francisco, San Francisco, CA, United States of America; Virginia Commonwealth University Medical Center, UNITED STATES

## Abstract

Alpha-1 adrenergic receptors mediate adaptive effects in the heart and cardiac myocytes, and a myocyte survival pathway involving the alpha-1A receptor subtype and ERK activation exists in vitro. However, data in vivo are limited. Here we tested A61603 (*N*-[5-(4,5-dihydro-1*H*-imidazol-2-yl)-2-hydroxy-5,6,7,8-tetrahydronaphthalen-1-yl]methanesulfonamide), a selective imidazoline agonist for the alpha-1A. A61603 was the most potent alpha-1-agonist in activating ERK in neonatal rat ventricular myocytes. A61603 activated ERK in adult mouse ventricular myocytes and protected the cells from death caused by the anthracycline doxorubicin. A low dose of A61603 (10 ng/kg/d) activated ERK in the mouse heart in vivo, but did not change blood pressure. In male mice, concurrent subcutaneous A61603 infusion at 10 ng/kg/d for 7 days after a single intraperitoneal dose of doxorubicin (25 mg/kg) increased survival, improved cardiac function, heart rate, and cardiac output by echocardiography, and reduced cardiac cell necrosis and apoptosis and myocardial fibrosis. All protective effects were lost in alpha-1A-knockout mice. In female mice, doxorubicin at doses higher than in males (35–40 mg/kg) caused less cardiac toxicity than in males. We conclude that the alpha-1A-selective agonist A61603, via the alpha-1A adrenergic receptor, prevents doxorubicin cardiomyopathy in male mice, supporting the theory that alpha-1A adrenergic receptor agonists have potential as novel heart failure therapies.

## Introduction

Cardiac myocyte alpha-1 adrenergic receptors (α1-ARs) mediate crucial adaptive functions in the heart, including physiological hypertrophy, contractility, survival signaling, ischemic preconditioning, and protection against multiple injuries, by activating multifactorial signaling cascades, as reviewed [1,2,3]. Among the 3 α1-AR subtypes (α1A, α1B, and α1D), rodent and human myocytes have the α1A and α1B [4], and most data implicate the α1A in adaptive effects [5,6,7,8,9,10,11]. However, it is unknown if an α1A-AR agonist can treat ardiomyopathy in vivo.

A61603 (*N*-[5-(4,5-dihydro-1*H*-imidazol-2-yl)-2-hydroxy-5,6,7,8-tetrahydronaphthalen-1-yl]methanesulfonamide), is a potent and selective imidazoline agonist for the α1A [12]. Selectivity for the α1A is important for potential clinical use, since the α1D might cause constriction of human epicardial coronary arteries [13], and the role of the α1B remains uncertain.

The anthracycline doxorubicin (DOX, Adriamycin) is a highly effective, widely used treatment for many pediatric and adult cancers, but has the severe, dose-limiting adverse effect of cardiotoxicity [14]. Previously, we found that adenoviral expression of the α1A receptor in myocytes from the α1-AR knockout (KO) reduced necrosis and apoptosis after DOX in vitro, through an ERK signaling mechanism [8], and that A61603 prevented DOX-induced apoptosis in vivo, detected by an MRI approach [9].

Here we first verified the potency of A61603 in myocyte ERK activation and protection from DOX in vitro. We identified a very low A61603 dose, 10 ng/kg/d, which activated cardiac ERK in vivo but did not increase blood pressure (BP), the classic role of α1-ARs [15]. Using this low dose, we tested the hypothesis that the beneficial effects of α1A stimulation with A61603 would translate to treatment of anthracycline cardiomyopathy in a mouse model in vivo.

We find that A61603 can prevent DOX cardiomyopathy in male mice, that this effect requires the α1A receptor, and that mechanisms include less cell death and fibrosis. We also observe that female mice are less susceptible to DOX cardiotoxicity, contrary to female humans, who are said to be more susceptible to anthracyclines [16,17]. The data support the idea that α1A agonists might translate to new therapy for heart failure (HF).

## Materials and Methods

### Animals

Female Sprague-Dawley rats with newborn litters were from Charles River; litters were used on arrival, and mothers were euthanized. Adult C57Bl/6J male and female mice age 14–18 weeks were from Jackson Laboratory, or our laboratory where wild type (WT) and α1A-AR knockout (KO) mice were produced [15]. All studies were reviewed and approved by the IACUC of the San Francisco VA Medical Center. Our institution is accredited by the American Association for the Accreditation of Laboratory Animal Care and has an Animal Welfare Assurance on file with the NIH Office for Laboratory Animal Research. Mice were housed in groups in sterile micro-isolator cages with food and water available ad libitum. Chow was Envigo Harlan Teklad, 2019 for breeders and 2016 for other mice. Light was on a 12 h schedule, temperature was 68–79°F, and humidity was 30–70%. Animals were inspected daily, and a licensed veterinarian was available at all times. Euthanasia was by cardiectomy under deep anesthesia with isoflurane. Criteria for euthanasia were signs of distress, including inactivity, poor grooming, hunched posture, weight loss >20% body weight (BW), or if heart rate (HR) was less than 200 bpm. For osmotic minipump and telemeter implantation, anesthesia was with isoflurane 1.5–3% in oxygen, and analgesia was with local bupivacaine 0.25% and systemic buprenorphine 5–100 μg/kg s.c.

### Drugs

Drugs were as follows: A61603 (*N*-[5-(4,5-dihydro-1*H*-imidazol-2-yl)-2-hydroxy-5,6,7,8-tetrahydronaphthalen-1-yl]methanesulfonamide hydrobromide) (Tocris #1052); ABT-866 (N-[3-(1H-imidazol-4-ylmethyl)phenyl]ethanesulfonamide) (synthesized by Synterys, Union City, CA); cirazoline (Tocris now R&D # 0888); dobutamine (Fisher Scientific, ICN#15978010); doxorubicin (Tocris #2252); epinephrine (Sigma #E4375); methoxamine (Sigma #M6524); midodrine (MP-Bio #155717); norepinephrine (Sigma #N5785); oxymetazoline (Tocris #1142); phenylephrine (Sigma #P-6126); propranolol (Fluka #82066); and ST-1059 (Toronto Research Chemicals #S686650).

### Neonatal rat ventricular myocyte isolation and culture

Neonatal rat ventricular myocytes (NRVMs) were isolated with trypsin digestion and mechanical dissociation, preplated to remove contaminating non-myocardial cells, and counted, with a yield of viable myocytes (excluding trypan blue) of approximately 3.5–4 million per neonatal heart. Cells at 500 per mm^2^ were plated overnight in 35 mm dishes in MEM with 5% calf serum and bromodeoxyuridine (BrdU), then maintained at 37°C with 1% CO2, in serum-free MEM with Hank’s salts, supplemented with penicillin, vitamin B12, BrdU, human transferrin 10 μg/ml, bovine insulin 10 μg/ml, and BSA 1 mg/ml [18,19]. Plating efficiency was approximately 25–30% of viable myocytes plated.

### Adult mouse ventricular myocyte isolation and culture

Adult mouse ventricular myocytes (AMVMs) were isolated following a detailed protocol [20]. Briefly, hearts were removed from WT and α1A-KO adult male mice under deep anesthesia with isoflurane, after heparin 100 IU in PBS i.p. to prevent coagulation of blood in the coronary arteries. Myocytes were obtained by perfusion with collagenase in nominally calcium-free buffer with butanedione monoxime (BDM) 10 mM, followed by mechanical dissociation and gradual calcium reintroduction, with a yield of 1–2 million cells per heart. Sixty thousand rod-shaped myocytes were plated onto laminin-coated 35 mm dishes in Minimal Essential Medium (MEM) with Hank’s salts, supplemented with calf serum 10%, BDM 10 mM, and penicillin 100 U/ml. After 2 h for myocyte attachment, cells were maintained in serum free MEM with Hank’s salts, supplemented with BSA 1 mg/ml and penicillin at 37°C with 2% CO2.

### ERK activation

For ERK activation (phosphorylation), NRVMs or AMVMs after 1 day in culture were pretreated 10 min with propranolol (PROP) 200 nM, then treated 5 min with A61603 or vehicle at 37°C, 2% CO2. Ventricular myocardium was collected after 7 d infusion in vivo with A61603 or vehicle. Samples were lysed in 1.5X SDS sample buffer containing Complete Mini protease and phosphatase inhibitor cocktails 2 and 3, snap-frozen, and stored at -80°C. For immunoblot, samples in SDS sample buffer were boiled for 5 min at 100°C; equal numbers of cells (25,000 NRVMs, 12,000 AMVMs) or equal amounts of ventricular protein (Bradford, 20 μg) per lane were separated on 12.5% Criterion Tris-HCl SDS-PAGE gels; and proteins were transferred to nitrocellulose membranes. Membranes were blocked with non-fat milk 5% in TBS-T, then incubated overnight at 4°C with 1:1,000 primary antibody in BSA-Tween 5%. Antibodies were total ERK1/2 (Cell Signaling #9102) and phospho-ERK1/2 (Cell Signaling #4370, phospho Thr202/Tyr204-p44/42 MAPK, D12.14.4E, XP® Rabbit mAb). Secondary antibody in milk 5% in TBS-T (anti-rabbit IgG, HRP-linked antibody, Cell Signaling #7074) was incubated 1 h at RT, and bands were developed using SuperSignal West Dura ECL reagent (Thermo Scientific #34076). Images were captured with the ChemiDoc XRS system (Bio-Rad), and analyzed with QuantityOne software version 4.6.9 (Bio-Rad).

### Toxicity assay in AMVMs

For toxicity assay, AMVMs the day after isolation were pre-treated 10 min with A61603 or vehicle, and PROP 200 nM, then DOX 20 μM or MEM vehicle. The next day cells were assayed using the MTT CellTiter Proliferation Assay protocol (ATCC #30-1010K), and absorbance of the purple formazan product indicating viable mitochondrial dehydrogenase activity was measured in triplicate aliquots at 570 nm on a GloMax-Multi+ Microplate reader (Promega). Blank wells were media plus MTT and Detergent Reagents, without cells.

### Drug delivery in vivo

Alzet osmotic mini-pumps (Durect) were used for continuous, subcutaneous (s.c.) drug delivery in vivo. Pump model #1002 has a mean pumping rate 0.25 μl per hour, and a maximum duration 14 days. Pumps were filled as stated by the package instructions; mice were anesthetized with isoflurane; and pumps were implanted s.c. between the scapulae.

### Blood pressure (BP) by telemetry

Telemetry BP was measured in awake, unrestrained 12 w male mice using PA-C10 BP transmitters (Data Sciences International, DSI). Under anesthesia with isoflurane (induction 3% in oxygen 100%, maintenance 1.5%), the pressure-sensing catheter was positioned in the aortic arch via the left carotid artery, and the transmitter body was placed s.c. along the left flank. Mice were recovered 7 d before an experiment. The Acquisition and Analysis programs of the Dataquest ART software (DSI) were used to collect and analyze BP data. For BP with continuous s.c. dosing, mice were housed throughout in the same room. BP was acquired with either 24 h continuous recording (12 h rest 6 am to 6 pm; 12 h activity 6 pm to 6 am), or twice-daily sessions during periods of rest (2 to 3 pm) or activity (12 to 1 am). Baseline BP was established over 4 d, after which pumps were implanted with A61603 (10 ng/kg/d or 10 μg/kg/d), NE (2.5 mg/kg/d), or vehicle (vitamin C 100 μM in 0.9% NaCl). After pump implantation, BP measurements began immediately for the 24 h continuous recording, or the following d for the twice-daily recordings, for a total of 7 d. BP parameters were averaged, and compared with baseline values.

### Echocardiography (echo)

Conscious, gently restrained mice underwent echo using an Acuson S2000 (Siemens) with a 5-14-MHz multi-dimension matrix transducer [21]. Ventricular dimensions were captured using 2-dimensional M-mode, with measurements taken at the mid-left ventricle (LV), just distal to the mitral valve leaflet plane, and acquired from five consecutive cardiac cycles in the long axis view. The echocardiographer was blinded to the genotype and treatments of all mice. Fractional shortening (FS), cardiac output (CO), stroke volume (SV), and ventricular volumes were calculated as follows [22]:

FS (%) = [(LVIDd–LVIDs)/LVIDd] x 100
CO (ml/min) = (SV×HR)/1000
SV (μl) = LVEDV−LVESV
LVEDV (μl) = [7/(2.4+LVIDd)] x LVIDd^3^
LVESV (μl) = [7/(2.4+LVIDs)] x LVIDs^3^

Where LVIDd, LV internal diameter in diastole; LVIDs, LV internal diameter in systole; HR, heart rate (from echo); LVEDV, LV end diastolic volume; LVESV, LV end systolic volume.

### Acute doxorubicin (DOX)-induced cardiomyopathy

Mice were given DOX in 250 μl saline vehicle intraperitoneal (i.p.), males 25 mg/kg or females 15–40 mg/kg. Prior to DOX, picking numbers randomized males to continuous s.c. dosing with A61603 10 ng/kg/d or vehicle (100 μM vitamin C) by osmotic mini-pump, with a ratio in WT mice of 2 vehicle to 1 A61603. Pumps were filled with drug or vehicle and coded by an independent person, and the code was not broken until the end of the experiment, so that all operators were blinded. Mice were monitored daily, and sacrificed at 7 days, or earlier if there were signs of distress, including inactivity, poor grooming, hunched posture, weight loss >20% BW, or if HR was less than 200 bpm, a sign of severe cardiomyopathy. Echocardiograms (echos) were done at baseline in some mice, and in all mice on the day of sacrifice. At sacrifice, BW was recorded, blood was collected, and organs and tibia were harvested.

### Blood analysis for cardiomyocyte death

Blood was collected by left ventricle (LV) apical puncture with a 20-gauge needle. Whole blood was allowed to clot, centrifuged at 13,000 rpm for 10 min, and the serum supernatant was snap-frozen, and stored at -80°C. Creatine kinase (CK) activity was measured in non-hemolyzed serum samples using the CK UV-rate kit (Stanbio Labs).

### Heart histology for apoptosis and fibrosis

Hearts were perfused in situ via the LV apex with KCl 60 mM to arrest in diastole, and fixed by paraformaldehyde 4%. After paraffin embedding, 6 μm sections were cut from LV base to apex every 100 μm, with an average of 5 sections per step collected per heart.

Sections were stained for fibrosis using the Picrosirius Red Stain kit (Polysciences, Inc.) and for apoptosis using the APO-BrdU™ TUNEL Assay Kit (Molecular Probes/Invitrogen). Random fields of the LV myocardium were imaged under phase microscopy at 40x magnification using a SPOT Imaging Solutions camera and software. Images were analyzed with ImageJ.

### Data analysis

Results are presented as mean ± SE. Significant differences (*p*<0.05) were tested using one-way ANOVA with Newman-Keuls multiple comparison test for more than two groups, or Student’s unpaired t-test for two groups (GraphPad Prism v4.0c). Survival data were analyzed by Kaplan-Meier curves. Normal distribution for continuous variables was confirmed using the D’Agostino and Pearson omnibus test.

## Results

### A61603 is the most potent α1-agonist in ERK activation in myocytes

**Fig 1:**
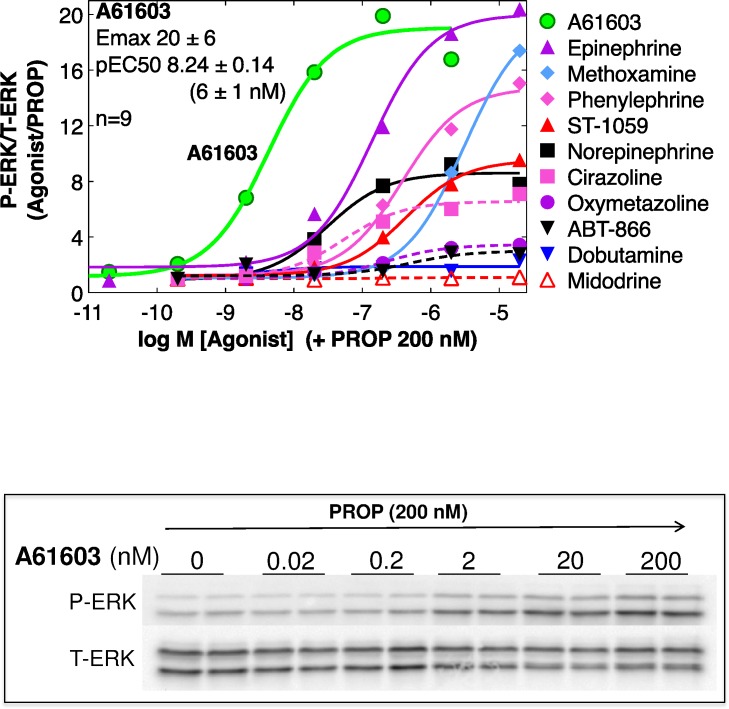
A61603 potently activates ERK in cultured neonatal rat ventricular myocytes (NRVMs). ERK activation was quantified by immunoblot as the ratio of phospho- to total-ERK (P-ERK/T-ERK) after 10 min with the beta-blocker propranolol (PROP) 200 nM, then 5 min with various α1-agonists or vehicle. Dose-response curves normalized to vehicle+PROP were analyzed by non-linear regression (N = 3–8 independent cultures, each dose in duplicate in each experiment). A61603 blots are shown. Table 1 has Emax and EC50 for each agonist.

Previously, we defined an α1A-ERK survival pathway using adenoviral expression in α1-KO cardiac myocytes [8]. Here we tested 10 α1-agonists for ERK activation in cultured NRVMs. The β-AR antagonist propranolol, which does not itself activate ERK (unpublished data), was present throughout, since some α1-agonists also activate β-ARs (e.g. norepinephrine, epinephrine, phenylephrine). **Fig 1** shows that A61603 was the most potent in ERK activation (EC50 6 nM), and was highly efficacious (20-fold increase). **Table 1** summarizes the pEC50 and Emax values for ERK activation, and also shows the binding affinities for each agonist at recombinant α1-subtypes, from the literature. The calculated binding selectivity for the α1A over the α1B or α1D shows that A61603 is the most selective of all agonists for the α1A (Table 1). These data confirmed that A61603 activated the α1A-ERK pathway with high potency and efficacy.

**Table 1:** Alpha-1-AR agonist ERK potency, binding affinity, and subtype selectivity.

	ERK Activation in NRVMs			Alpha-1-AR Binding Affinity (pKi)			Binding Selectivity		
	pEC50	Emax	n	Alpha-1A	Alpha-1B	Alpha-1D	A/B	A/D	B/D
A61603	8.24 ± 0.14	20.5 ± 6.1	9	7.74 ± 0.11 (7)	5.68 ± 0.08 (5)	5.85 ± 0.01 (5)	115	78	0.7
Dobutamine	7.65 ± 0.28	2.8 ± 0.7	5	7.00 (1)	na	Na	0.4	0.1	0.2
Norepinephrine	7.57 ± 0.11	8.6 ± 1.3	7	5.45 ± 0.18 (17)	5.67 ± 0.13 (19)	6.83 ± 0.16 (13)	0.6	0.04	0.07
Cirazoline	7.25 ± 0.12	6.6 ± 0.1	3	6.79 ± 0.14 (5)	6.40 ± 0.17 (4)	6.94 ± 0.27 (4)	2.5	0.7	0.3
Epinephrine	7.16 ± 0.12	19.6 ± 5.9	6	5.45 ± 0.14 (13)	5.82 ± 0.15 (15)	6.44 ± 0.10 (11)	0.4	0.1	0.2
ABT-866	6.71± 0.32	2.9 ± 0.7	3	6.84 ± 0.02 (3)	6.06 ± 0.003 (3)	6.55 ± 0.003 (3)	6.1	1.9	0.3
Oxymetazoline	6.63 ± 0.09	3.5 ± 0.1	3	7.69 ± 0.09 (20)	6.62 ± 0.05 (21)	6.07 ± 0.08 (15)	12	42	3.5
Phenylephrine	6.42 ± 0.11	15.0 ± 5.2	5	5.36 ± 0.17 (14)	5.28 ± 0.14 (14)	6.24 ± 0.16 (10)	1.2	0.1	0.1
Midodrine/ST-1059	6.40 ± 0.05	9.7 ± 1.1	3	5.63 ± 0.21 (4)	5.14 ± 0.04 (4)	5.60 ± 0.18 (4)	3.1	1.1	0.3
Methoxamine	5.54 ± 0.04	20.0 ± 6.9	4	4.52 ± 0.18 (14)	3.54 ± 0.17 (15)	4.65 ± 0.15 (10)	9.4	0.7	0.1

Dose-response curves for 10 alpha-1-agonists, in duplicate dishes for each dose in each experiment (n), were generated for ERK activation (5 min agonist treatment), in the presence of the beta-blocker propranolol 200 nM.

Potency (pEC50) and efficacy (Emax) for each experiment were from non-linear regression analysis.

Binding affinity (pKi) for each agonist at recombinant alpha-1-AR subtypes is from the literature (n = reports).

The agonist selectivity for each alpha-1-receptor subtype was calculated as A/B = (10^(pKi A—pKi B)), and similarly for A/D and B/D.

For midodrine/ST-1059, functional data are reported for the active compound ST-1059; and binding data are for the parent compound midodrine.

Values are mean ± SE.

na = not available.

Agonists are ordered according to the ERK pEC50.

### A61603 activates ERK and protects adult cardiac myocytes from DOX in vitro, via the α1A

**Fig 2:**
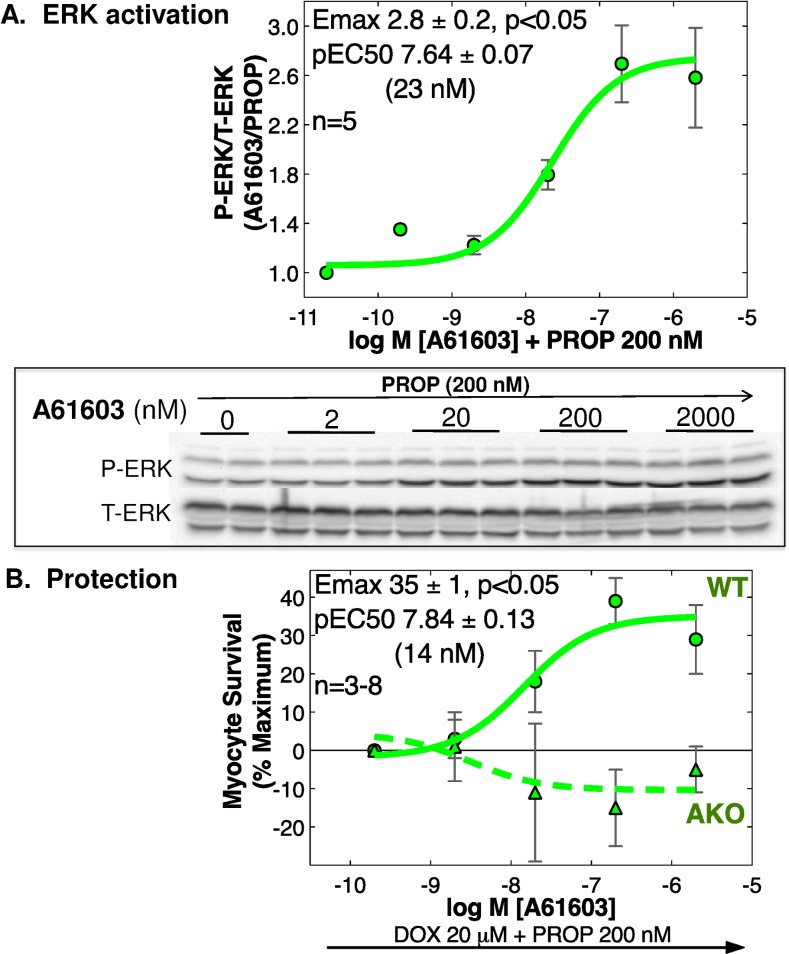
A61603 activates ERK and protects WT adult mouse ventricular myocytes (AMVMs) from DOX toxicity in vitro, via the α1A. **(A)** ERK activation in cultured AMVMs was quantified by immunoblot as the ratio of phospho- to total-ERK (P-ERK/T-ERK) after 10 min with the beta-blocker PROP 200 nM, then 5 min with A61603 or vehicle. Dose-response curves normalized to vehicle+PROP were analyzed by non-linear regression (n = 5 cultures from separate hearts, each in duplicate; values are mean ± SE). Emax is significantly different from 1.0 by 95% confidence limits (p<0.05). Representative blots are shown. (**B**) WT or α1A KO cultured AMVMs were treated overnight with DOX 20 μM and PROP 200 nM, in the presence of varying doses of A61603 or vehicle. Myocyte survival by MTT assay is shown as mean ± SE, and was analyzed by non-linear regression and 2-way ANOVA, relative to PROP alone at 100% and DOX+PROP at 0%; n = 3–8 cultures from separate hearts, each in duplicate or triplicate. WT pEC50 and Emax are shown. Emax is significantly different from 1.0 by 95% confidence limits (p<0.05).

In cultured adult mouse ventricular myocytes (AMVMs), A61603 activated ERK, and protected myocytes from DOX toxicity (**Fig 2**). The EC50 values for these 2 effects were very similar (23 and 14 nM, Fig 2). The lower potency and efficacy of A61603 in AMVM ERK activation in comparison with NRVMs (Fig 1) is consistent with the 10-fold lower density of α1A-ARs in AMVMs [2]. In α1A KO myocytes, A61603 did not prevent DOX toxicity, confirming that A61603 was working specifically at the α1A receptor on cardiac myocytes to confer a protective effect (Fig 2B). These data showed that A61603 activated the myocyte ERK survival pathway defined previously [8].

### A61603 activates cardiac ERK in vivo at subpressor dosing

**Fig 3:**
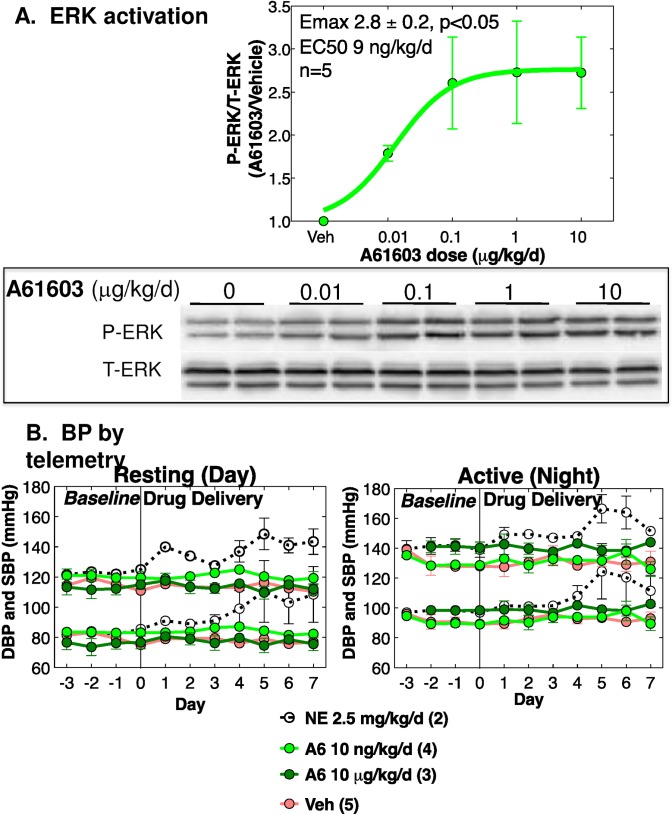
A61603 activates ERK in vivo at subpressor dosing. **(A)** Normal male WT mice age 10–12 w were treated with A61603 (0.01–10 μg/kg/d) or vehicle (100 μM vitamin C) continuously for 7 d via s.c. osmotic mini-pump, and cardiac ERK activation was quantified by immunoblot as the ratio of P-ERK/T-ERK. Results are for the cytosolic fraction of ventricular homogenates, which contained approximately 75% of total- and phospho-ERK. The A61603 dose-response curve was normalized to vehicle, and analyzed by non-linear regression (N = 5 mice per dose, mean ± SE). Emax is significantly different from 1.0 by 95% confidence limits (p<0.05). A blot is shown below. (**B**) Baseline BP was recorded for 4 d, then A61603 (10 ng/kg/d and 10 μg/kg/d) or vehicle were infused continuously for 7 d by osmotic pump. Systolic BP (upper traces) and diastolic BP (lower traces) are shown during (**left**) periods of rest (2–3 pm) and (**right**) periods of activity (12–1 am). Numbers of mice are indicated. A hypertensive dose of NE (2.5 mg/kg/d) was a positive control.

We infused normal adult male WT C57Bl6J mice with A61603 (0.01 to 10 μg/kg/d) by s.c. osmotic minipump, and quantified cardiac ERK activation by immunoblot after 7 d. A61603 increased ventricular phospho-ERK by 2.8-fold, with an EC50 9 ng/kg/d (**Fig 3A**). To measure BP in awake, unrestrained mice, we inserted telemetry catheters in the aorta and infused A61603 10 ng/kg/d or 10 μg/kg/d or vehicle for 7 days, after 4 days baseline recording. From 24 h recording, we identified periods of maxium res (2–3 PM) or activity (12–1 PM) for detailed analysis. A61603 had no effect of systolic or diastolic BP in resting or active mice (**Fig 3B**). Previously, we found that A61603 increases BP with an EC50 300 ng/kg given acutely intravenous [15], as expected for an α1-AR agonist [23]. Thus, A61603 activated cardiac ERK at dosing that was far below that required to increase BP.

### A61603 improves survival in an acute cardiotoxic DOX HF model

Among multiple models of DOX cardiotoxicity, acute high-dose models are used to test efficacy of potential therapies [24]. We did a trial in an acute, high-dose DOX model to test for beneficial effects of A61603 in vivo.

**Fig 4:**
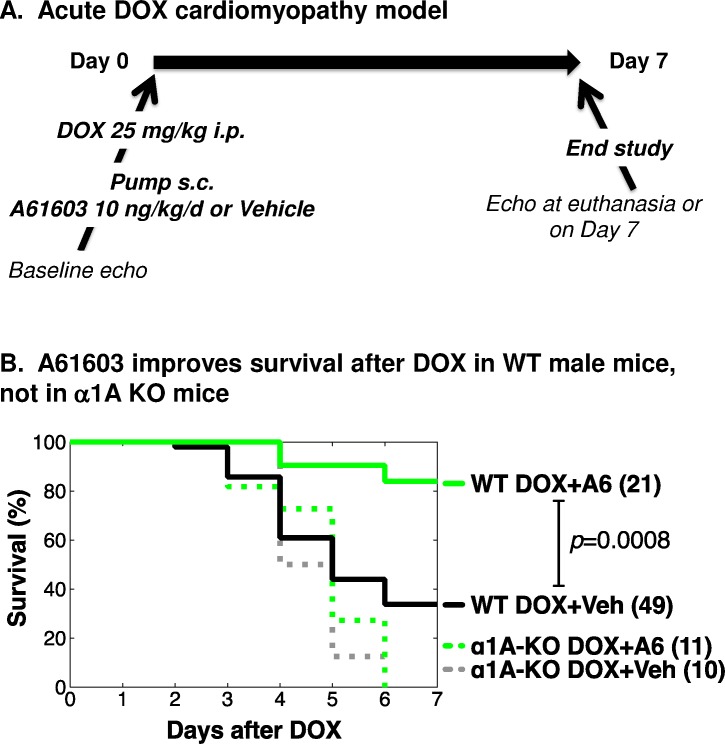
Low-dose A61603 increases survival of WT male mice in a doxorubicin (DOX) cardiomyopathy model, but not in α1A-KO mice. (**A**) Young male WT and α1A-KO mice were randomized to A61603 10 ng/kg/d (A6) or vehicle (100 μM vitamin C, Veh) via s.c. osmotic mini-pump. Immediately after pump implantation, mice were injected with a single dose of DOX 25 mg/kg i.p. Mice were monitored daily, and euthanized for HF per protocol or on day 7. Echo was done prior to treatment in some mice, and on the day of sacrifice in surviving mice. Operators were blinded to genotype and treatment. (**B**) Kaplan-Meier 7-day survival curves, with numbers of mice indicated.

Young male mice were given DOX in a single dose, 25 mg/kg i.p. Seventy WT mice and 21 α1A-KO mice were randomized to continuous s.c. infusion by osmotic mini-pump of A61603 or vehicle (100 μM vitamin C). Immediately after pump implantation, mice were injected once with DOX. A61603 dosing was 10 ng/kg/d, which activates cardiac ERK but does not change BP (Fig 3). Echo was done at baseline in some mice, and then in all mice at euthanasia, when dictated by signs of distress or bradycardia, or at study end after 7 days, according to the protocol shown in **Fig 4A**. All operators were blinded to genotype and treatment.

In WT male mice after DOX, A61603 improved survival to day 7 to 84%, from 34% with vehicle (*p*<0.0008) (**Fig 4B**). In α1A-KO mice, A61603 did not improve survival after DOX, and mortality was 100% by day 6 regardless of treatment, worse than WT mice (Fig 4B). Therefore, the beneficial survival effect of the α1A-agonist A61603 during DOX cardiotoxicity required the α1A receptor, indicating an on-target effect.

### A61603 preserves cardiac function after acute high-dose DOX

**Fig 5:**
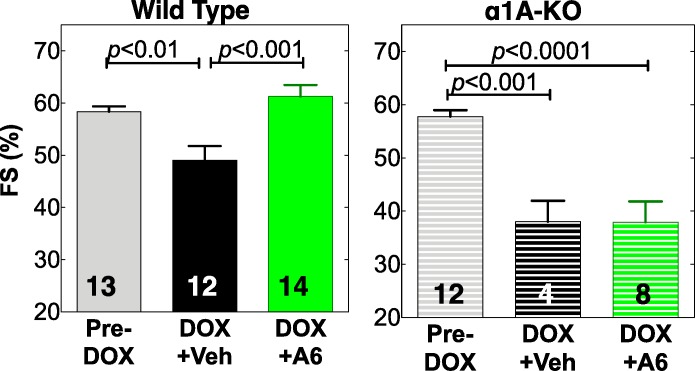
A61603 preserves cardiac function after DOX in WT male mice, but not in α1A-KO mice. Mice given DOX were treated with A61603 or Vehicle as in Fig 4. Values are fractional shortening (FS) by echo, mean ± SE, n = mice, *p* by one-way ANOVA with Newman-Keuls multiple comparisons test.

We used FS by echo in awake mice to quantify LV function after DOX. Following DOX injection, FS fell in surviving vehicle-treated WT mice, from 58% pre-DOX to 49% at euthanasia for HF or at day 7 (n = 12, p<0.01) (**Fig 5, Table 2**). A61603 prevented this decline in cardiac function in WT mice, with FS 61% post-DOX (n = 14, p<0.001 vs. DOX+Veh) (Fig 5). In α1A KO mice, A61603 did not prevent the drop in FS (58% pre-DOX to 38% post-DOX) (Fig 5). It was not possible to do a terminal echo on all studied mice, since many died unexpectedly during the night.

**Table 2:** Echocardiography of male mice treated with DOX.

	WILD TYPE				Alpha-1A KO			
	Pre-DOX	Post-DOX			Pre-DOX	Post-DOX		
		Vehicle	A61603	A6/Veh		Vehicle	A61603	A6/Veh
mice (n)	13	7	6		12	4	8	
FS (%)	58 ± 4	49 ± 3 (12)++	61 ± 2 (14)	1.20***	58 ± 1	38 ± 4+++	38 ± 4+++	0.99
LVIDd (mm)	3.1 ± 0.1	2.5 ± 0.1+++	2.7 ± 0.1++	1.08	3.0 ± 0.1	2.3 ± 0.3+	2.3 ± 0.2++	0.98
LVIDs (mm)	1.3 ± 0.1	1.4 ± 0.1	1.3 ± 0.1	0.89	1.2 ± 0.1	1.4 ± 0.1	1.4 ± 0.2	1.01
LVPWd (mm)	0.9 ± 0.0	1.0 ± 0.1	0.9 ± 0.1	0.92	0.9 ± 0.0	1.1 ± 0.1	1.1 ± 0.1	1.02
LVPWs (mm)	1.6 ± 0.1	1.2 ± 0.1++	1.4 ± 0.1	1.15	1.5 ± 0.0	1.1 ± 0.1++	1.3 ± 0.1++	1.12
IVSd (mm)	0.9 ± 0.1	1.00 ± 0.1	0.9 ± 0.1	0.95	0.9 ± 0.0	0.9 ± 0.1	1.0 ± 0.1	1.05
IVSs (mm)	1.7 ± 0.1	1.4 ± 0.1	1.6 ± 0.1	1.11	1.7 ± 0.0	1.4 ± 0.2+	1.4 ± 0.1+	1.06
SV (μV (±l)	34 ± 1	17 ± 2+++	23 ± 3+++	1.37*	31 ± 2	15 ± 1++	14 ± 3+++	0.74
HR (bpm)	663 ± 12	300 ± 79+++	513 ± 77+	1.71*	657 ± 11	252 ± 16+++	214 ± 47+++	0.85
CO (ml/min)	22 ± 1	6 ± 2+++	13 ± 2+++	2.27**	20 ± 1	4 ± 2+++	3 ± 1+++	0.79
CO/BW (ml/min/g)	0.8 ± 0.0	0.2 ± 0.1+++	0.7 ± 0.1++	3.04***	0.8 ± 0.0	0.2 ± 0.1+++	0.1 ± 0.0+++	0.59

WT or alpha-1A-KO mice average age 16.5 weeks had DOX 25 mg/kg i.p., then were treated with A61603 10 ng/kg/d or Vehicle by mini-pump.

2D-guided M-mode echo was done Pre-DOX in some mice, and Post-DOX in all mice, either at euthanasia for HF or at 7 days.

Values are mean ± SE, with *n* (mice) given or in parentheses.

The *n* for Post-DOX FS is higher, since this was the only value recorded in an initial series of mice.

*p* by ANOVA with Newman-Keuls multiple comparisons test: A61603 vs. Vehicle

*** = <0.001

** = <0.01

* = <0.05

Post-DOX vs. Pre-DOX

+++ = <0.001

++ = <0.01

+ = <0.05.

Pre-DOX values for WT vs. alpha-1A-KO did not differ *(p*>0.1).

To evaluate the A61603 mechanisms involved in preserving cardiac function measured by FS after DOX, LV dimensions were examined. LVIDd was markedly reduced after DOX in all mice (Table 2), consistent with cardiac atrophy, as seen in mice and humans with anthracycline cardiomyopathy [25,26,27]. Reduced heart weight at study end supported the conclusion of cardiac atrophy (below). WT mice treated with A61603 had trends toward a larger LVIDd, smaller LVIDs, and greater systolic thickness of the LV posterior wall (LVPWs) and interventricular septum (IVSs), all consistent with less atrophy and improved systolic function (Table 2). Most of these trends were absent in α1A KO mice (Table 2).

### A61603 preserves hemodynamics after acute high-dose DOX

**Fig 6:**
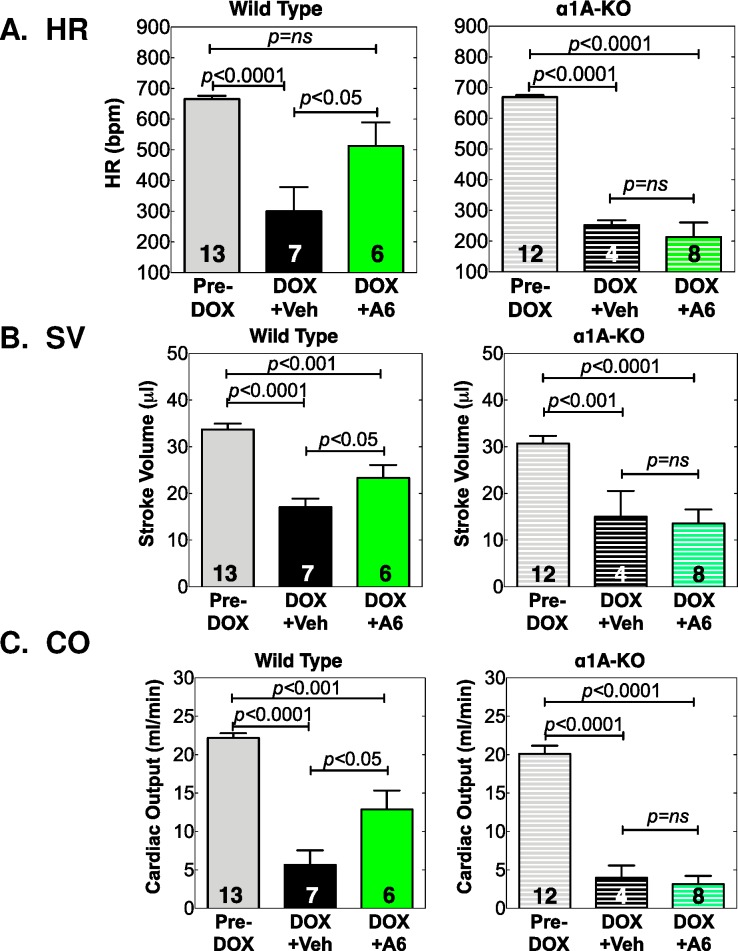
A61603 preserves hemodynamics in male mice after DOX, via the α1A. Mice given DOX were treated with A61603 or Vehicle as in Fig 4. Shown are (**A**) HR, (**B**) SV, and (**C**) CO, all from echo, for each treatment group and genotype. Values are mean ± SE, n = mice, *p* by one-way ANOVA with Newman-Keuls multiple comparisons test.

Interestingly, HF in mice is associated with bradycardia. WT mice given vehicle with DOX had a 55% decrease in HR vs. Pre-DOX, whereas WT mice treated with A61603 had only a 23% decrease, a significant improvement vs. vehicle (*p*<0.05, Table 2, **Fig 6**). A61603 did not preserve HR in α1A KO mice (Table 2, Fig 6). Similarly, SV, the amount of blood ejected with each beat, was reduced by 50% vs. Pre-DOX in WT mice given vehicle with DOX, whereas the drop in SV with DOX was only 33% with A61603 treatment (p<0.05 vs. vehicle). The improved SV with A61603 was lost in α1A KO mice (Table 2, Fig 6). Together, the reduced HR and SV caused by DOX drastically reduced CO in WT mice treated with vehicle by 73%, whereas the decrease in CO was only 41% in WT mice treated with A61603 (*p*<0.01 vs. vehicle). Again this protective effect of A61603 on CO was lost in the α1A KO (Table 2, Fig 6). Preservation of hemodynamics by A61603 was even more impressive when CO was normalized to body weight (BW), to derive a cardiac index (88% of Pre-DOX with A61603, 25% of Pre-DOX with vehicle, *p*<0.001 A61603 vs. vehicle, Table 2). Overall, these data suggested that mortality after DOX was due to reduced cardiac function, and that A61603 improved survival by improving function, an effect that required the α1A receptor.

### A61603 prevents cardiac cell death and myocardial fibrosis in the acute DOX model

**Fig 7:**
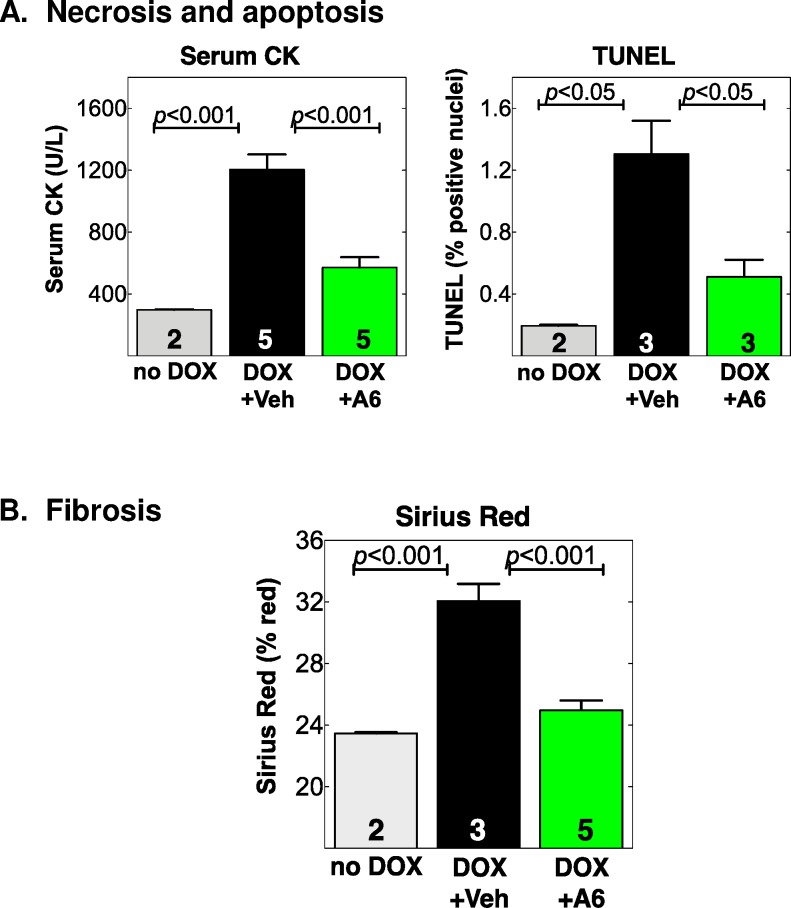
A61603 prevents cardiac cell death and myocardial fibrosis after DOX in WT male mice. Male mice were treated with DOX or saline injection, and randomized to A61803 or Vehicle, as in Fig 4. Shown are indices at end of study for (**A**) Cardiac myocyte necrosis measured by serum CK activity in blood collected by LV puncture, and TUNEL staining for apoptosis in LV sections; and (**B**) LV myocardial fibrosis by sirius red staining. Number (n) is hearts; *p* by one-way ANOVA with Newman-Keuls multiple comparisons test.

To identify potential cell mechanisms whereby A61603 preserved cardiac function and hemodynamics, we quantified indices of cardiac cell death and fibrosis in WT mice. DOX increased serum creatine kinase (CK) activity, an assay for myocyte necrosis, by 4-fold at euthanasia vs. no DOX, and A61603 prevented this increase (p<0.001 vs. vehicle) (**Table 3, Fig 7**). DOX increased LV nuclear TUNEL staining, an index of cardiac apoptosis, by 7-fold vs. no DOX, and A61603 prevented this increase (p<0.05 vs. vehicle) (Fig 7). DOX increased LV sirius red staining, a measure of myocardial fibrosis, by 1.4-fold, and A61603 prevented this increase (p<0.0001 vs. vehicle) (Fig 7). These data confirmed and extended our prior finding that A61603 in the DOX model reduced fibrosis and apoptosis detected by an MRI approach [9], and suggested that A61603 prevention of cardiac cell death and fibrosis could explain preservation of LV function and overall hemodynamics.

**Table 3:** Pathology of male mice treated with DOX.

	Wild Type			Alpha-1A KO		
	Vehicle	A61603	A6/Veh	Vehicle	A61603	A6/Veh (%)
mice (n)	6	5		4	7	
BW Pre-DOX (g)	27.0 ± 0.7	26.2 ± 0.6	0.97	26.2 ± 0.5	26.3 ± 0.8	1.00
BW Post-DOX (g)	22.7 ± 0.6	23.2 ± 0.5	1.02	22.0 ± 0.4	20.8 ± 0.5	0.95
Change in BW (g)	-4.3 ± 0.6	-3.0 ± 0.8	0.70	-4.2 ± 0.5	-5.5 ± 0.4	1.31
% Change in BW	-16 ± 3	-11 ± 3	0.72	-16 ± 2	-21 ± 1	1.30*
VW (mg)	104 ± 6	113 ± 9	1.09	103 ± 8	99 ± 5	0.97
VW/BW	4.6 ± 0.2	4.9 ± 0.3	1.06	4.7 ± 0.3	4.8 ± 0.2	1.03
Tibia length (mm)	16.8 ± 0.1	17.5 ± 0.2	1.04*	17.5 ± 0.3	17.3 ± 0.2	0.99
VW/TL	6.2 ± 0.4	6.5 ± 0.5	1.04	5.8 ± 0.4	5.8 ± 0.3	0.98
Serum CK (U/L)	1204 ± 98 (5)	571 ± 67 (5)	0.47***	nd	nd	
TUNEL (% positive nuclei)	1.30 ± 0.22 (3)	0.51 ± 0.11 (3)	0.39*	nd	nd	
Fibrosis (% area)	32.1 ± 1.1 (3)	25.0 ± 0.6 (5)	0.78***	nd	nd	

WT or alpha-1A-KO mice were treated as in Fig 1, with DOX 25 mg/kg/i.p., then A61603 10 ng/kg/d or vehicle, and pathology was done 7 days after DOX, or at euthanasia for HF.

Values are mean ± SE, with *n* (mice or hearts) given or in parentheses.

BW and VW are given only for mice that had measurements both Pre-DOX and Post-DOX.

*p* by Student’s t-test; A61603 vs. Vehicle:

*** = <0.001

* = <0.05. nd = not done.

### Effect of A61603 on body weight and heart weight in the acute DOX model

WT mice treated with DOX lost body weight (BW) over the study, and A61603 tended to reduce the weight loss (11% loss A61603 vs. 16% vehicle, *p* = 0.3), a trend that was not seen in α1A KO mice (Table 3). Similarly, combined right and left ventricular weight (VW), VW/BW, and VW normalized to tibia length (TL) trended higher with A61603, and this trend was absent in α1A KO mice (Table 3). VW in mice given DOX and treated with vehicle was significantly less than in a comparable group of 20 mice not given DOX (104 ± 6 mg, n = 6 vs. 127 ± 3, n = 20, p<0.05). However, there was no difference when VW was normalized to BW (4.6 ± 0.2, n = 6 DOX vs. 4.8 ± 0.1, n = 20 no DOX). Thus there was cardiac atrophy in proportion to the loss of BW.

### Female mice are less susceptible to acute DOX cardiotoxicity

**Fig 8:**
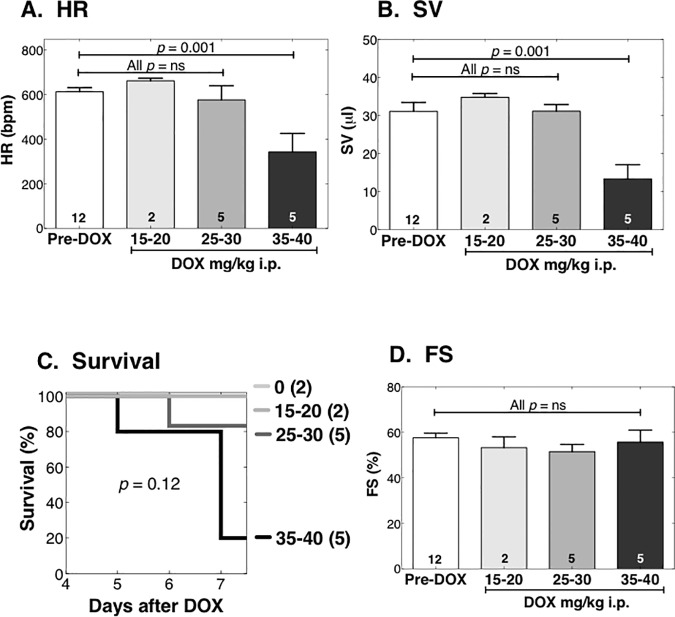
Young female mice are resistant to DOX cardiac toxicity. Female WT mice age 15 weeks were randomized to vehicle or a single dose of DOX 15–40 mg/kg i.p. Mice were monitored daily for 7 days, and euthanized for HF per protocol or on day 7. Echo was done prior to treatment, and on the day of sacrifice. Operators were blinded. The highest DOX dose reduced HR (**A**) and SV (**B**), and tended to reduce survival (**C**), but DOX did not change FS (**D**). *p* by one-way ANOVA with Newman-Keuls multiple comparisons test.

To assess the clinical relevance to females of this acute DOX cardiomyopathy model, we studied a cohort of young female mice in the same model used for males (Fig 4A). We used DOX doses (mg/kg i.p.) of 15–20, 25–30, and 35–40. In the young females, no dose reduced survival or FS significantly; only the 35–40 mg/kg dose reduced BW, LVIDd, HR, and SV, and thereby reduced CO and CI (**Fig 8, Table 4)**. Overall, young female mice were resistant to DOX cardiotoxicity in comparison with young males, requiring a much higher dose (35–40 mg/kg) for a phenotype even approaching that in young males after DOX 25 mg/kg. The 35–40 mg/kg dose is roughly 105–120 mg/m^2^, or twice that of the human single-dose equivalent of 60 mg/m^2^. Therefore, this model does not mimic the increased susceptibility of human females to DOX [16,17], and we did not pursue additional studies of acute high-dose DOX in young female mice.

**Table 4:** Echocardiography and pathology of WT female mice treated with DOX.

	Pre-DOX	DOX dose (mg/kg i.p.)		
		15–20	25–30	35–40
mice (n)	12	2	5	5
FS (%)	58 ± 2	53 ± 5	52 ± 3	56 ± 5
LVIDd (mm)	3.0 ± 0.1	3.2 ± 0.1	3.1 ± 0.1	2.1 ± 0.3+++
LVIDs (mm)	1.3 ± 0.0.08	1.5 ± 0.2	1.5 ± 0.1	1.0 ± 0.2
LVPWd (mm)	0.8 ± 0.06	0.9 ± 0.1	0.9 ± 0.1	1.2 ± 0.2+
LVPWs (mm)	1.5 ± 0.05	1.5 ± 0.1	1.4 ± 0.1	1.6 ± 0.1
IVSd (mm)	0.9 ± 0.04	0.8 ± 0.1	0.8 ± 0.1	1.1 ± 0.1
IVSs (mm)	1.6 ± 0.06	1.6 ± 0.1	1.4 ± 0.1	1.6 ± 0.1
SV (microl)	31± 2	35 ± 1	31 ± 2	13 ± 4+++
HR (bpm)	613 ± 19	663 ± 12	577 ± 64	343 ± 83+++
CO (ml/min)	19 ± 1	23 ± 0	18 ± 2	5 ± 3+++
CO/BW (ml/min/g)	0.9 ± 0.1	1.1 ± 0.0	0.8 ± 0.1	0.3 ± 0.1+++
BW (g)	21.7 ± 0.3	21.1 ± 0.2	21.0 ± 1.1	17.1 ± 1.0++
Change in BW (g)		-0.7 ± 0.2	-0.7 ± 1.1	-4.0 ± 1.0
% Change in BW		-3 ± 1	-5 ± 6	-24 ± 6
VW (mg)		103 ± 3	105 ± 6	83 ± 6#
VW/BW		4.9 ± 2.0	5.0 ± 0.4	4.7 ± 0.1
TL (mm)		17.0 ± 0.2	17.3 ± 0.2	17.3 ± 0.2
VW/TL		6.1 ± 2.0	6.1 ± 0.4	4.8 ± 0.3

WT female mice had baseline echo, then were treated with varying doses of DOX.

Mouse dosing was as follows (mg/kg i.p.): 15–20 (1 mouse 15, 1 mouse 20), 25–30 (3 mice 25, 2 mice 30), 35–40 (4 mice 35, 1 mouse 40).

Echo was repeated at euthanasia for distress or bradycardia or at 7 days, and pathology was done.

Values are mean ± SE, with *n* (mice) given.

*p* by ANOVA with Newman-Keuls multiple comparisons test: DOX dose vs. Pre-DOX

+++ = <0.001

++ = <0.01

+ = <0.05.

high DOX vs. low or medium DOX:

# = <0.05

## Discussion

The major new finding of this study is that an α1A-AR selective agonist, the imidazoline A61603, can prevent anthracycline-induced cardiomyopathy in male mice. We found that A61603 was the most potent among 10 α1-agonists in activating myocyte ERK, and identified a low dose of A61603, 10 ng/kg/d, that activated cardiac ERK in vivo, without changing BP. Infusion of this low A61603 after a single DOX dose improved survival and preserved cardiac function and hemodynamics. These beneficial effects could be explained by A61603-mediated protection from cardiac cell death and fibrosis, and protection from death was reproduced in myocytes in vitro. The protective effects of A61603 required the α1A receptor in vitro and in vivo, and α1A KO mice had increased mortality and worse function and hemodynamics with DOX. Overall, these data provide support for the use of α1A-selective agonists as HF therapies.

Myocyte death is a fundamental cause of anthracycline cardiomyopathy [28], and multiple cellular mechanisms are responsible, including abnormalities in transcription factors, myofibrils, calcium regulation, mitochondria, extracellular matrix, and formation of reactive oxygen species [28,29,30]. As an example, we find that a different α1A agonist protects cardiac mitochondria and ATP levels [31].

The signaling mechanisms involved in α1A protection from myocyte death include ERK activation, which we found is required for adenoviral expression of the α1A to rescue α1A/B KO cardiomyocytes from DOX-induced death [8]. We show here that A61603 potently activates this α1A-ERK survival pathway in cultured myocytes and also protects adult myocytes from DOX-induced death via the α1A (Figs 1 and 2). We also show that A61603 activates ERK in the normal heart In vivo with an EC50 9 ng/kg/d (Fig 3), suggesting that dosing in the cardiomyopathy model, 10 ng/kg/d, is activating ERK in myocytes. ERK activation by A61603 in the injury model in vivo is difficult to test directly, since, whereas the α1A receptor is expressed in myocytes but not nonmyocytes [39], ERK is activated robustly in nonmyocytes in cardiac injury [32], obscuring direct effects in myocytes.

ERK is a central myocyte protective kinase [33], and several potential cardioprotective pathways downstream of α1-ARs and/or ERK are identified, such as PKCɛ, p90 ribosomal S6 kinase, SOD, iNOS, anti-apoptotic Bcl proteins, adenosine, cyclooxygenase-2, GATA-4, and others [1,2,33]. Indeed, the ability of α1-ARs in general to activate pleiotropic signaling could explain their efficacy against multiple injuries, including oxygen radicals, ischemia-reperfusion, myocardial infarction, and pressure overload (review in [1,2,3]).

The current DOX model has limitations. The DOX dosing of 25 mg/kg in males is high, but was used in many prior studies [34,35,36,37,38], and is equivalent by allometric scaling to about 75 mg/m^2^ in a mouse [39], only 25% higher than a typical single human dose of 60 mg/m^2^. However, mortality is high in this model, most likely from the severe reductions in heart rate and stroke volume, producing a 75% decrease in cardiac index in vehicle-treated mice, vs. only 12% decrease with A61603 (Table 2). Humans do not typically have reduced heart rate in HF. Also unlike humans, where females are more susceptible to anthracyclines [16,17], female mice were resistant relative to males, as in other heart disease models [40]. We studied only young mice (14–18 weeks), equivalent to approximately 25 human years, and effects in aged males and females are unknown. DOX caused weight loss, which was reduced but not prevented by A61603 (Table 3), consistent with poor feeding from low cardiac index and/or systemic toxicity, such as liver or bone marrow damage [41,42,43,44], which can also be seen in humans. Alternative chronic dosing models might better simulate cardiac toxicity with less systemic toxicity [45]. Overall, this high dose model might be more relevant for the acute DOX toxicity observed in about 10–30% of patients [46,47,48].

These limitations not withstanding, the data do show clearly that a small molecule α1A agonist can prevent cardiac injury and improve cardiac function and hemodynamics, at a very low dose that does not change BP. Besides this report, several lines of evidence support the translation of α1A-agonist cardioprotection to human HF. α1-ARs levels and regulation are similar in mouse and human heart [4], and A61603 activates protective ERK signaling in human LV myocardium ex vivo [11]. Although α1-AR levels represent roughly 10% of the AR population in non-diseased hearts, with β-ARs at 90%, β-AR down-regulation in HF increases the proportion of α1-ARs to nearly 25% [2,4,49]. In addition, as β-AR function deteriorates in HF, α1-mediated inotropy can become similar to β-AR [50]. Antagonism of α1-AR signaling with nonselective α1-blockers can cause HF and death, as shown in the ALLHAT and V-HeFT trials [51,52]. Altogether, these studies provide indirect human proof of concept for α1A agonist therapy.

Potential concerns regarding α1A-agonist therapy exist, and the main arguments have been reviewed [1]. One concern would be increased BP. However, the benefit of A61603 is seen at 10 ng/kg per day, dosing that does not change BP (Fig 3), and is far below the EC50 of 300 ng/kg for increasing BP with IV bolus dosing [15]. Thus an ample therapeutic window exists for cardioselective dosing.

As a note of caution, several other potential drugs are reportedly cardioprotective in anthracycline cardiotoxicity models, such as thrombopoietin [25], erythropoietin [53], melatonin [36], CB1 cannabinoid receptor antagonists [54], granulocyte colony-stimulating factor [55], and ErbB2 [56], so that proof of concept in other forms of cardiomyopathy will be required to advance the concept of an α1A agonist as a drug to treat HF.

## Conclusions

In conclusion, the α1A-AR selective agonist A61603, at a very low subpressor dose, prevents cardiomyopathy induced by an anthracycline in male mice. Protection in males requires the α1A receptor and involves ERK activation and prevention of cardiomyocyte death. The model is less useful in female mice. These findings confirm and extend previous observations of cardioprotection by α1A-agonist therapy, and support the developing model of α1A agonists as novel therapies for HF.
